# Potassium augments growth, yield, nutrient content, and drought tolerance in mung bean (*Vigna radiata* L. Wilczek.)

**DOI:** 10.1038/s41598-024-60129-z

**Published:** 2024-04-23

**Authors:** Mohammad Rafiqul Islam, Umakanta Sarker, Mohammad Golam Azam, Jamil Hossain, Mohammad Ashraful Alam, Riaz Ullah, Ahmed Bari, Nazmul Hossain, Ayman El Sabagh, Mohammad Sohidul Islam

**Affiliations:** 1 Agronomy Division, Regional Agricultural Research Station, Bangladesh Agricultural Research Institute (BARI), Ishwardi, Pabna, 6620 Bangladesh; 2 Department of Genetics and Plant Breeding, Faculty of Agriculture, Bangabandhu Sheikh Mujibur Rahman Agricultural University, Gazipur, 1706 Bangladesh; 3 Pulses Research Centre, BARI, Ishwardi, Pabna, 6620 Bangladesh; 4 Plant Breeding Division, Spices Research Centre, BARI, Bogura, Bangladesh; 5 Department of Pharmacognosy, College of Pharmacy, King Saud University, Riyadh, Saudi Arabia; 6 Department of Pharmaceutical Chemistry, College of Pharmacy, King Saud University, Riyadh, Saudi Arabia; 7 Department of Agronomy, Iowa State University, Ames, IA 50010 USA; 8 Department of Agronomy, Faculty of Agriculture, Kafrelsheikh University, Kafrelsheikh, 33156 Egypt; 9 Department of Agronomy, Hajee Mohammad Danesh Science and Technology University, Dinajpur, Bangladesh

**Keywords:** Plant stress responses, Abiotic

## Abstract

Uneven rainfall and high temperature cause drought in tropical and subtropical regions which is a major challenge to cultivating summer mung bean. Potassium (K), a major essential nutrient of plants can alleviate water stress (WS) tolerance in plants. A field trial was executed under a rainout shelter with additional K fertilization including recommended K fertilizer (RKF) for relieving the harmful impact of drought in response to water use efficiency (WUE), growth, yield attributes, nutrient content, and yield of mung bean at the Regional Agricultural Research Station, BARI, Ishwardi, Pabna in two successive summer season of 2018 and 2019. Drought-tolerant genotype BMX-08010-2 (G1) and drought-susceptible cultivar BARI Mung-1 (G2) were grown by applying seven K fertilizer levels (KL) using a split-plot design with three replications, where mung bean genotypes were allotted in the main plots, and KL were assigned randomly in the sub-plots. A considerable variation was observed in the measured variables. Depending on the different applied KL and seed yield of mung bean, the water use efficiency (WUE) varied from 4.73 to 8.14 kg ha^−1^ mm^−1^. The treatment applying 125% more K with RKF (KL_7_) under WS gave the maximum WUE (8.14 kg ha^−1^ mm^−1^) obtaining a seed yield of 1093.60 kg ha^−1^. The treatment receiving only RKF under WS (KL_2_) provided the minimum WUE (4.73 kg ha^−1^ mm^−1^) attaining a seed yield of 825.17 kg ha^−1^. Results showed that various characteristics including nutrients (N, P, K, and S) content in stover and seed, total dry matter (TDM) in different growth stages, leaf area index (LAI), crop growth rate (CGR), root volume (RV), root density (RD), plant height, pod plant^−1^, pod length, seeds pod^−1^, seed weight, and seed yield in all pickings increased with increasing K levels, particularly noted with KL_7_. The highest grain yield (32.52%) was also obtained from KL_7_ compared to lower K with RKF. Overall, yield varied from 1410.37 kg ha^−1^ using 281 mm water (KL_1_; well-watered condition with RKF) to 825.17 kg ha^−1^ using 175 mm water (KL_2_). The results exhibited that the application of additional K improves the performance of all traits under WS conditions. Therefore, mung beans cultivating under WS requires additional K to diminish the negative effect of drought, and adequate use of K contributes to accomplishing sustainable productivity.

## Introduction

Grain legumes contribute a significant role in the world economy through improved nutritional safety and soil health, especially in developing countries as they are rich in protein sources and nitrogen-fixing ability to the soil^1,2^. Mung bean (*Vigna radiata* L. Wilczek) subjected to one of the major annual grain legume crops^3^, accounts for 5% of the total world production of pulses^4^. However, global mung bean production is threatened by drought stress that leads to a reduction in growth and yield, particularly in tropical and subtropical regions where scarcity of water due to uneven rainfall is a common phenomenon^5^. High solar radiation and large temperature swings during the mung bean growing season (March–May) act as a double whammy, intensifying drought stress on the crop^6^. Also, the temperatures of more than 35 °C and straight solar radiation owing to global climate change are confirming injurious to summer sown mungbean that experiences suppression of growth in different developmental parts^7^. The spectrum of plant reactions to drought varies widely across different crops and stages of growth, shaped by the severity and persistence of water scarcity^8^.

Drought stress during critical growth phases significantly hampers mungbean growth and yield. It initially reduces stomatal conductance, stem elongation, leaf size, and root growth, consequently reducing nutrient uptake and resulting in a decline in grain yield^9^. This stress adversely affects yield components such as pod and seed numbers, along with individual seed weight^10^. Furthermore, it lowers plant height, dry weight, chlorophyll content, and essential nutrient levels like nitrogen (N), phosphorus (P), potassium (K), and sulfur (S)^11^. Drought also disrupts mineral uptake and N fixation in legumes^12,13^. Seed filling, a crucial stage for plant development, is compromised by drought and heat stress, triggering premature senescence and shortening the seed-filling period^14^. These stressors interfere in assimilate remobilization and cause alterations in protein levels and amino acid concentrations within seeds^15^.

Drought stress leads to significant changes in the internal chemical bonding and reduces the yield by up to 70%^16^. Besides, tolerance to adverse environments is very difficult, because of complex interactions between stress factors and different physio-biochemical, and molecular occurrences motivating plant enlargement and expansion^17^. Drought disturbs the development of plant phenology^18^, growth^19^, leaf area^20^, and productivity^21^ by alteration of various bio-physiological activities^22^. These activities mimicked drought conditions by inducing osmotic stress^23^ and overproduction of reactive oxygen species (ROS)^24^, ultimately leading to oxidative damage, a natural consequence of stress^25^. Osmotic stress in plants changes morphology^26^, protein^27^, membrane damage^28^, nutrient imbalance^29^, many enzymatic antioxidants^24^non-enzymatic antioxidants^30–34^, stomatal closer, and opening^35^ which can manage the adverse impact of abiotic stresses. It also hampers the plant water relation^20,22^ and nutrient uptake^36^ resulting in low growth and yield response,

Nutritional deficiency can retard plant growth and development, cause plant damage, and ultimately lead to yield reduction. To attain maximum yield and quality, it is necessary to supply adequate nutrients in regard to crop demands^37^. On the contrary, suitable genotypes/verities and good crop management including proper fertilizer management are responsible for high productivity in mung bean^38^. Potassium assumes a crucial role, both directly and indirectly, among applied nutrients, particularly under various stress conditions, and it a vital part in important plant mechanisms like photosynthesis, respiration, synthesis of protein, and stimulation of enzymes, which are strongly correlated with the overall growth and yield of crops^39,40^, as well as their resistance against the different diseases and pests^41^. Additionally, K contributes to the preservation of water absorption and osmotic potential, exerts a positive influence on stomatal closure^42^, enhances WUE^43^, and improves the water relations of the plant^44^ and these combined effects contribute to improve the tolerance of plants to water stress (WS)^45^. However, the K level in our soils is being depleted day by day due to practices of intensive cropping systems as well as the use of modern high-performance crop varieties, so far there is no alternative basis to restock it into the soil. The use of substantial amounts of K is noted as a decreasing factor in WS environments, and its consumption has positive effects in alleviating the adverse impacts associated with that condition^46^, It aids in facilitating the transport of different mineral compounds (NO_3_^−^, PO_3_^−^, Ca^2+^, Mg^2+^), amino acids, and the movement of water, and its uptake decreased when the K supply deviates from the optimal amount^47^. Hence, yield-limiting effects under WS conditions can be overcome through rising K application ^48^. To improve soil moisture, optimize nutrient content, and achieve a substantial yield in WS environments, it is essential to implement suitable management options including the adequate use of fertilizer.

Potassium fertilization significantly enhances the yield and quality of mung bean plants. Studies indicate that applying K fertilizers increases seed yield, protein content, and important yield components like thousand seed weight, plant height, seeds per pod, and first pod height^49,50^. Different levels of K application improve growth parameters, yield attributes, seed and haulm yields, and economic returns^51^. Optimal results, such as the highest protein content in seeds and maximum apparent K recovery efficiency, are achieved with specific K application rates^52^. Potassium plays a crucial role in protein formation, and the application of K has a positive impact on the growth of mungbean^53,54^. Overall, the proper use of K enhances productivity, quality, and nutrient content, and contributes to soil fertility maintenance in mung bean cultivation.

The preceding explanation strongly supports that drought poses a significant environmental challenge for summer-sown mungbean. Consequently, implementing strategic K management under WS conditions has the potential to greatly alleviate the negative impacts of drought stress on the development and productivity of mungbean. Additionally, the application of specific K application levels, particularly through a recommended K fertilizer (RKF) approach may improve WUE, total dry matter (TDM), leaf area index (LAI), crop growth rate (CGR), root development and nutrient absorption, collectively contributing to improved crop performance during drought stress. Furthermore, combining the use of tolerant genotypes with sufficient K fertilization synergistically works to mitigate the detrimental effects of WS on the yield of summer-sown mungbean plants. Thus, the study aims to observe the impacts of additional K fertilization for relieving the negative repercussions of drought stress specifically examining WUE, growth, nutrient content, yield attributes, and overall yield of summer mung bean cultivation.

## Results and discussion

### Effects of additional K fertilizer with RKF on WUE of mung bean under WS condition

**Figure 1:**
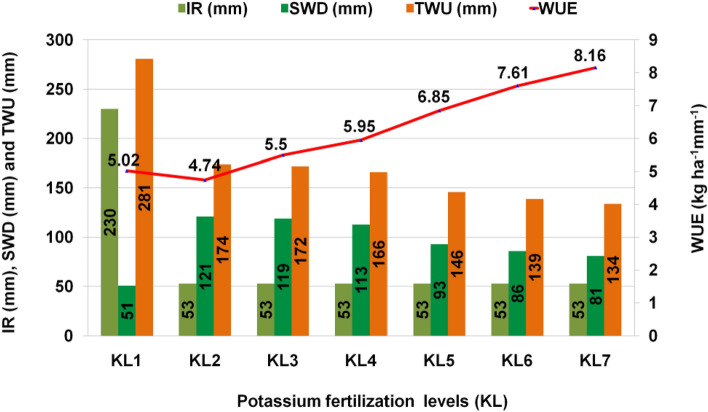
Effect of additional potassium (K) fertilization with RKF on the WUE of mung bean under WS (KL_1_ = WW + RKF (18 kg K ha^−1^); KL_2_ = WS + RKF; KL_3_ = WS + RKF + 25% additional K; KL_4_ = WS + RKF + 50% additional K; KL_5_ = WS + RKF + 75% additional K; KL_6_ = WS + RKF + 100% additional K; KL_7_ = WS + RKF + 125% additional K; *IR*  irrigation schedule, *SWD*  soil water depletion, *TWU* total water use, *WUE* water use efficiency.

The variations of additional K fertilization with RKF can be related to patterns of soil moisture depletion (Fig. 1)**.** The rate of soil water depletion (SWD) was lower (51 mm) under KL_1_ treatment than KL_2_ (121 mm), indicating to conservation of more soil moisture as compared to KL_1_. In addition, as the K level increases with RKF under WS condition (KL_3_-KL_7_), the rate of SWD decreases simultaneously (Fig. 1). The results indicate that K improves the soil moisture content. The highest total water use (281 mm) was exhibited in KL_1_ followed by the KL_2_ treatment plot (174 mm). The total water use was reduced in KL_2_ owing to declining soil moisture depletion. Based on different K fertilizer concentrations and the mung bean seed production under different water regimes, the WUE ranged from 4.74 to 8.16 kg ha^−1^ mm^−1^. With the highest WUE (8.16 kg ha^−1^ mm^−1^), the KL_7_ treatment produced a seed yield of 1093.60 kg ha^-1^. This observation may be attributed to the fact that the most efficient use of water coincided with corresponding water consumption, leading to increased seed yield. Likewise, KL_2_ treatment provided the minimum WUE (4.74 kg ha^−1^ mm^−1^) attaining a seed yield of 825.17 kg ha^−1^ followed by KL_1_ (5.02 kg ha^−1^ mm^−1^). Notably, soil moisture stress severely affects the grain yield of mung bean, affecting the WUE^5^, and heightened application of K (up to 175%) with recommended level alleviates this negative impact through overcoming soil moisture stress^55^.

### Effects of additional K fertilizer with RKF on growth traits of mung bean under WS condition

#### Total dry matter

**Figure 2:**
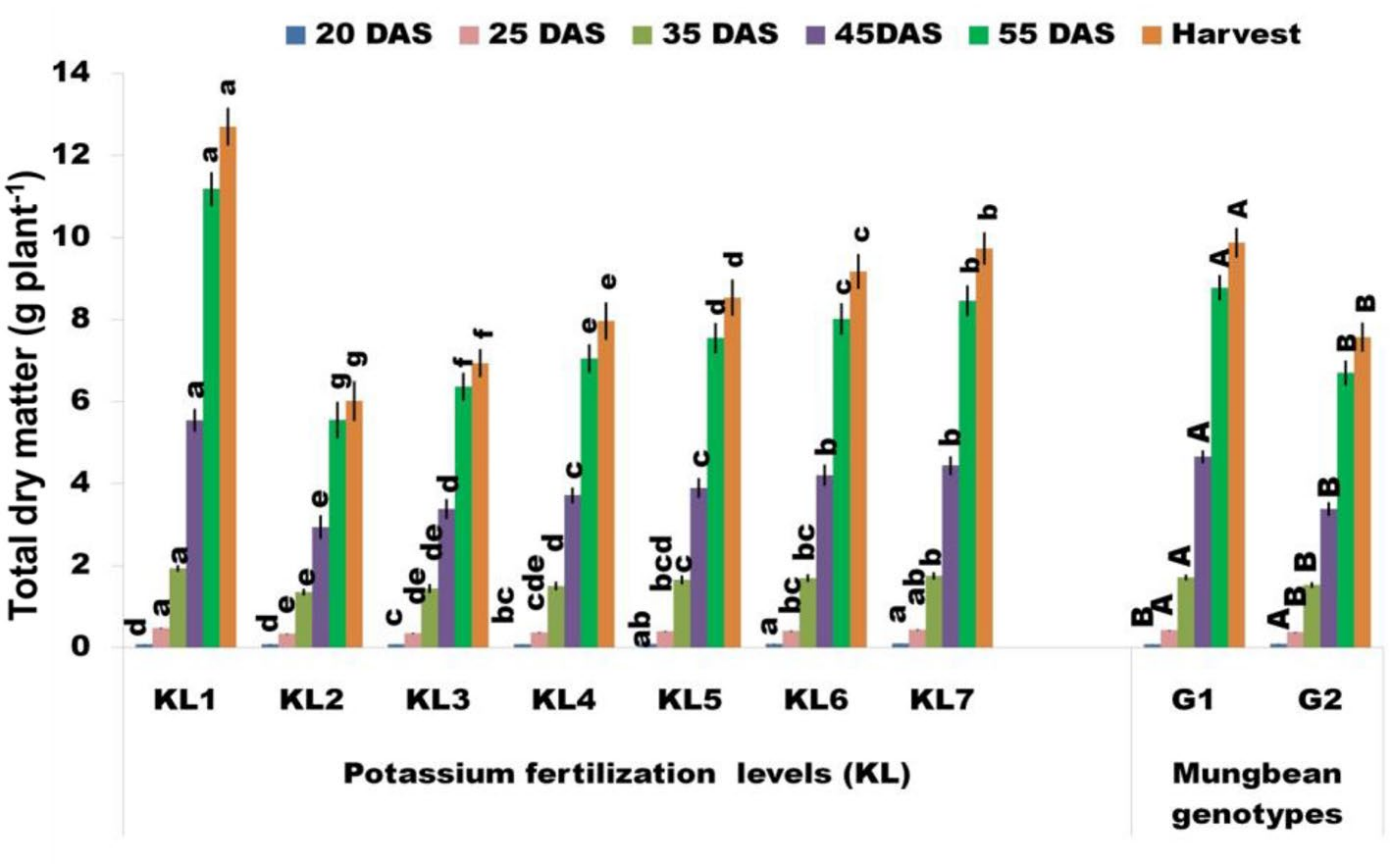
Effect of additional K fertilization on the TDM (g plant^−1^) at different pheno-phages of mung bean genotypes under WS (KL_1_ = WW + RKF (18 kg K ha^−1^); KL_2_ = WS + RKF; KL_3_ = WS + RKF + 25% additional K; KL_4_ = WS + RKF + 50% additional K; KL_5_ = WS + RKF + 75% additional K; KL_6_ = WS + RKF + 100% additional K; KL_7_ = WS + RKF + 125% additional K; Values within the bars having different letter(s) differed significantly at 5% level of probability as per LSD.

The amount of light energy that the leaves efficiently catch and convert to chemical energy determines how much TDM the plants produce. Enhancing mungbean productivity primarily hinges on optimizing TDM production and ensuring its appropriate partitioning to reproductive organs^18^. The TDM of both studied mung bean genotypes exhibited an increase with the advancement of plant maturity (Fig. 2). Under KL_2_, the TDM considerably declined as compared to KL_1_. However, the G1 genotype produced a higher TDM in response to WS across the respective growth stages, whereas it was comparatively lesser in G2 (Supplementary Table S1). This disparity could be attributed because the G1 genotype exhibited a higher LAI under WS conditions compared to that of G2, resulting in increased interception of light energy and subsequent conversion into higher TDM. The outcomes further exposed that TDM steadily increased with the rising K levels ranging from 25 to 125% (KL_3_–KL_7_) in comparison to KL_2_, and the highest increment of TDM accounting to 15, 25, 21, 31, 33, and 40%, were recorded with KL_7_ at the measured growth stages, respectively, surpassing those of KL_2_. The use of adequate K fertilizer exhibited a significantly positive relationship with dry matter accumulation, as reported in an earlier study^56^.

#### Leaf area index

**Figure 3:**
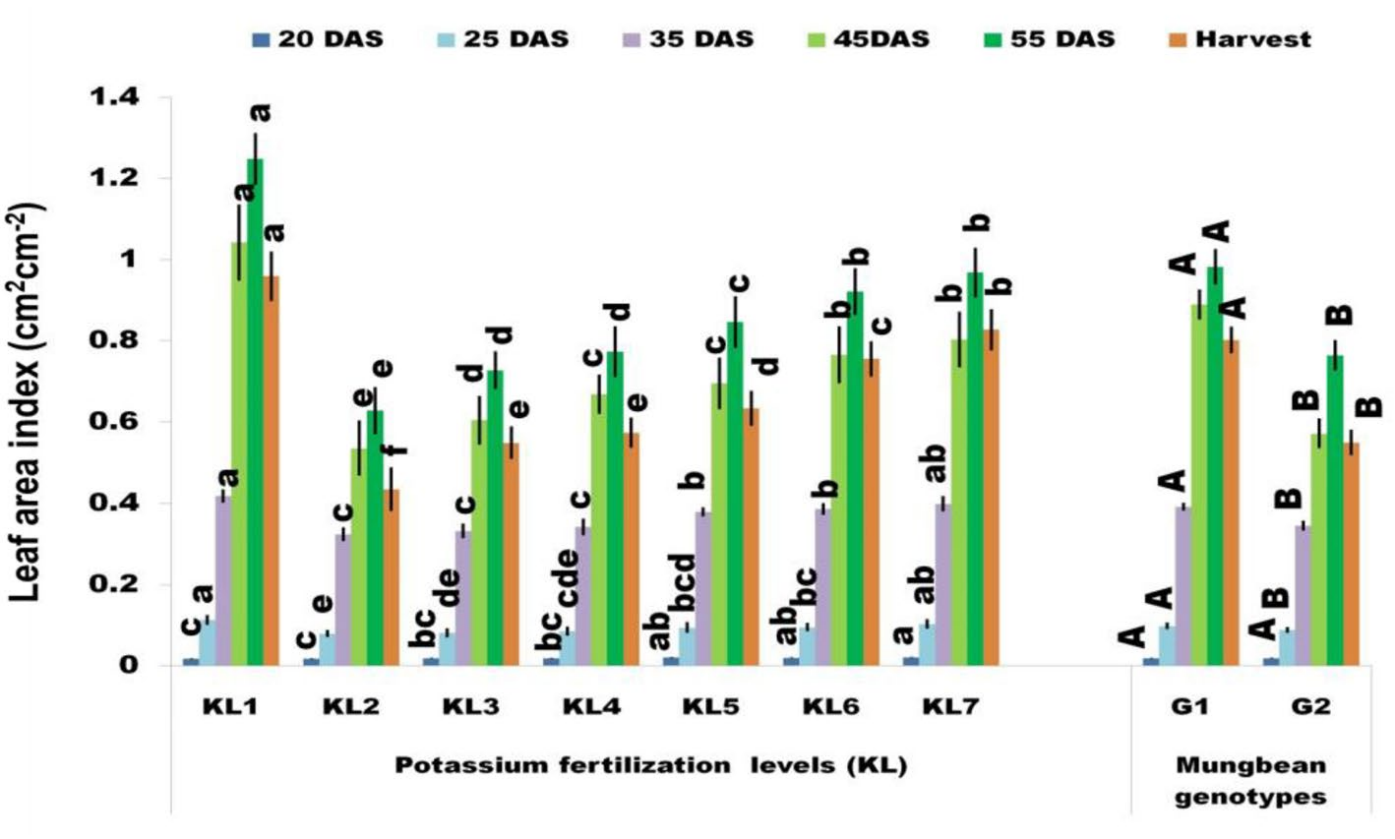
Effect of additional K fertilization on the LAI (cm^2^ cm^−2^) at different pheno-phages of mung bean genotypes under WS (KL_1_ = WW + RKF (18 kg K ha^−1^); KL_2_ = WS + RKF; KL_3_ = WS + RKF + 25% additional K; KL_4_ = WS + RKF + 50% additional K; KL_5_ = WS + RKF + 75% additional K; KL_6_ = WS + RKF + 100% additional K; KL_7_ = WS + RKF + 125% additional K; Values within the bars having different letter(s) differed significantly at 5% level of probability as per LSD.

One of the growth markers, LAI, regulates the overall absorption of light energy by plants and serves as an indicator of crop yield. The increase in dry matter production and the capture of solar radiation inside the canopy is closely associated with the rising LAI. Therefore, a decline in LAI is mirrored by a reduction in dry matter production ^57^. In a similar vein, Dwyer and Stewart^58^ found that crop light interception is significantly influenced by leaf area, which in turn has a significant impact on crop productivity. In the current investigation, the LAI grew steadily in both genotypes until the first pod color change stage (55 DAS), at which point it dramatically decreased as a result of certain leaves senescing. (Fig. 3). The mung bean genotypes significantly reduced the LAI under KL_2_ compared to KL_1_, and the reduction of LAI under KL_2_ might be due to the diminished WUE. However, the extreme LAI was recorded in the G1 genotype, and the least was noted in genotype G2 at the trifoliate stage of the leaf (20 DAS). The application of additional K fertilizer under stress conditions had a significant effect on the LAI. As regards, increasing K fertilizer by 25, 50, 75, 100, and 125% in stress treatment significantly increased the LAI and the maximum LAI was recorded with the highest K level (125%) at all the growth stages (Fig. 3). The adequate soil moisture after K treatment, leading to increased leaf area and dry weight, was the primary factor contributing to a discernible rise in LAI at the various growth phages. Genotype G1 performed better regarding LAI under KL_3_–KL_7_ treatment than G2, and KL_7_ in both genotypes produced the highest LAI from 20 DAS to the final harvest (Supplementary Table S2). WS is more prominent and affects crop phenology and leaf growth and development^18,59^ which ultimately turns or decreases the leaf area^60^. The results are also consistent with those of Nisha et al.^61^, who found that under stress circumstances at various development stages, WS significantly decreased the leaf area whereas K fertilizer enhanced the leaf area.

#### Crop growth rate

**Figure 4:**
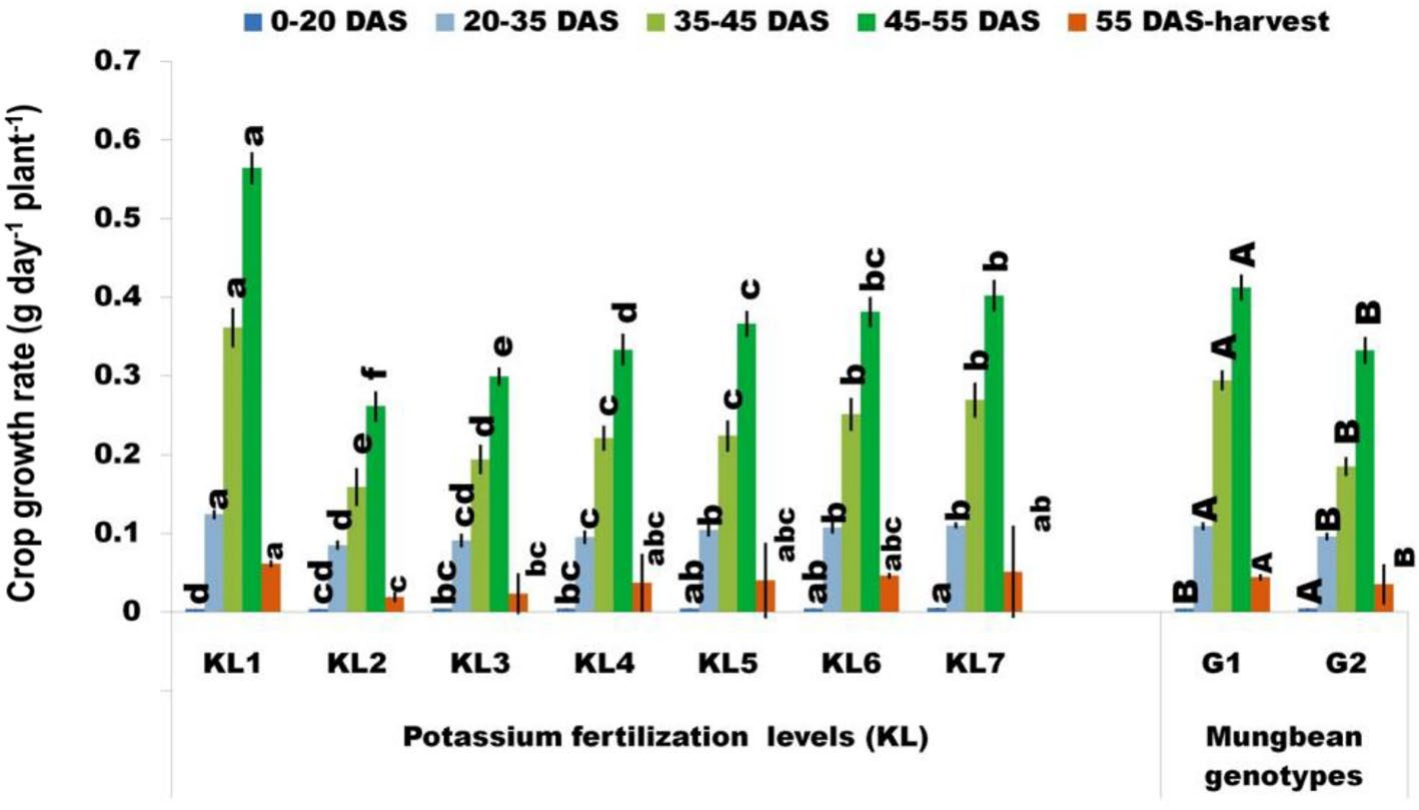
Effect of additional K fertilization on the CGR (g day^−1^ plant^−1^) at different pheno-phages of mung bean genotypes under WS (KL_1_ = WW + RKF(18 kg K ha^−1^); KL_2_ = WS + RKF; KL_3_ = WS + RKF + 25% additional K; KL_4_ = WS + RKF + 50% additional K; KL_5_ = WS + RKF + 75% additional K; KL_6_ = WS + RKF + 100% additional K; KL_7_ = WS + RKF + 125% additional K; Values within the bars having different letter(s) differed significantly at 5% level of probability as per LSD.

The dry matter buildup at various stages of development was used to determine CGR. In both genotypes, the CGR grew progressively up to 55 DAS before declining at harvest (Fig. 4). The halt of vegetative development, the loss and senescence of leaves, and the reduction in LAI at later growth stages are the causes of the CGR decline after 55 DAS. These factors may have also decreased the photosynthetic efficiency and, eventually, the dry matter accumulation rate^57^. The CGR under KL_2_ was reduced significantly compared to KL_1_ in mung bean genotypes at different stages of life span. The use of additional K fertilizer with KL_2_ (KL_3_–KL_7_) showed a considerable response to the CGR. The highest level of K (KL_7,_125%) produced the maximum CGR at all the growth stages ranging from 0.0046–0.0402 g day^−1^ plant^−1^, as compared to KL_2_. The favorable reaction of CGR to plants and the acceleration of photosynthetic activity might be the cause of the rise in CGR seen with increased K fertilizer. The growth of the G1 genotype was higher than G2 at all sampling dates in this study (Supplementary Table S3). After the trifoliate leaf stage (20 DAS) to 55 DAS, the higher CGR was recorded in the G1 genotype than in genotype G2. This might have been caused by higher LAI in G1 genotype which reflected higher TDM and CGR. The results are corroborated with earlier findings where noticed that a sufficient supply of K can augment the crop growth (total dry mass accumulation) under drought stress in contrast to lesser K levels^62^.

#### Root volume and Root density

**Figure 5:**
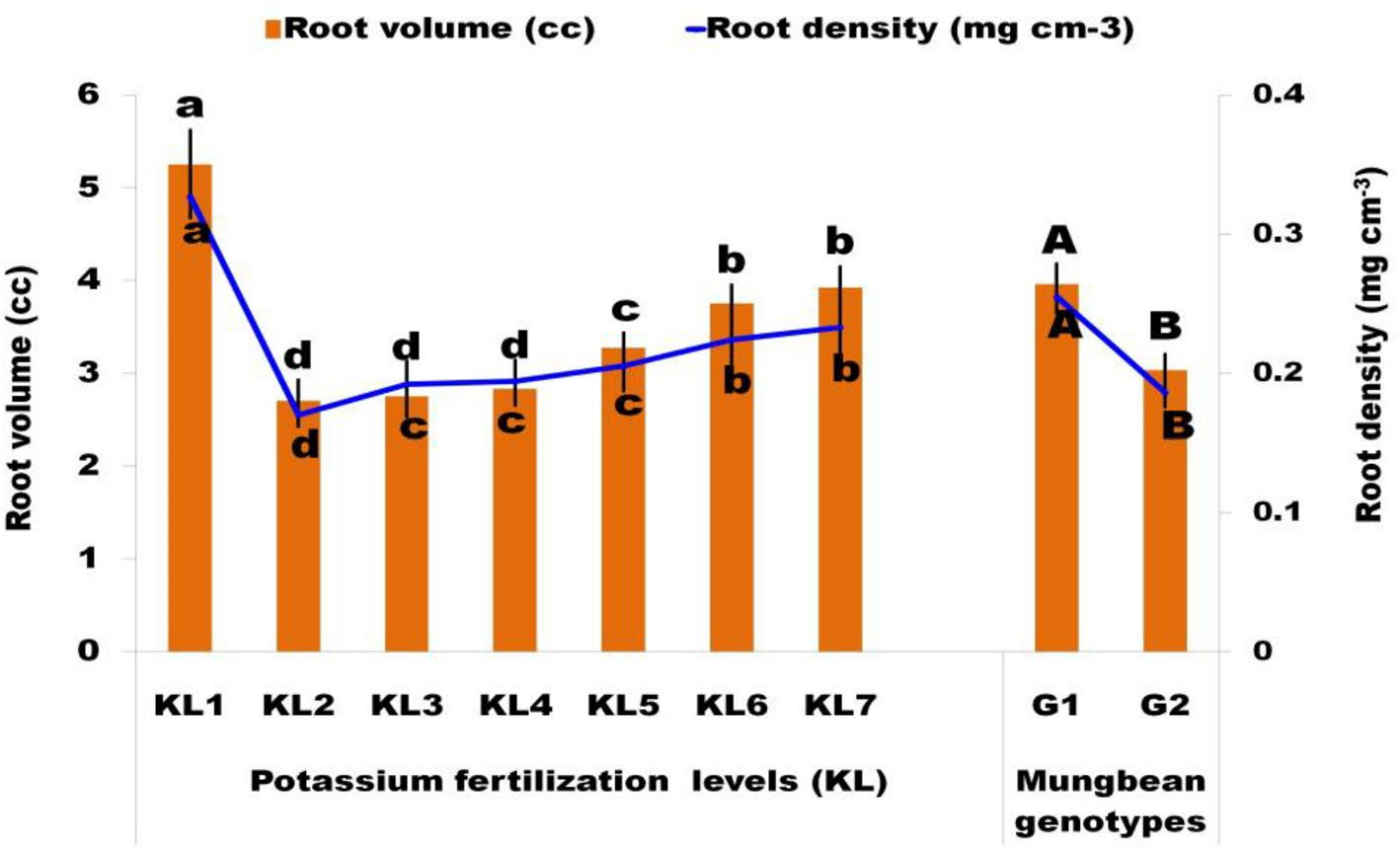
Effect of additional K fertilization on the RV and RD at harvest under mung bean genotypes under WS (KL_1_ = WW + RKF(18 kg K ha^−1^); KL_2_ = WS + RKF; KL_3_ = WS + RKF + 25% additional K; KL_4_ = WS + RKF + 50% additional K; KL_5_ = WS + RKF + 75% additional K; KL_6_ = WS + RKF + 100% additional K; KL_7_ = WS + RKF + 125% additional K; Values within the bars having different letter(s) differed significantly at 5% level of probability as per LSD.

Root growth (root volume and density) is a key factor for plant tolerance to WS as it is the main engine to meet the transpirational demand and plays a significant role in facilitating water availability to plants. The present study revealed that mung bean plants subjected to KL_2_ treatment exhibited a significant diminution in their root growth in the soil environment when compared to KL_1_. Additional K fertilization at the rate of 25, 50, 75, 100, and 125% (KL_3_–KL_7_) with KL_2_ considerably increased the root growth, and KL_7_ showed the highest root growth (Fig. 5). It might be due to increasing K fertilizer accelerating the development of lateral and secondary roots which leads to an increase in RV as well as density. Nisha et al.^61^ also showed that in mung beans grown under WS conditions, there was an increase in root development with the rise in K content. The results are also consistent with the findings of Dhole et al. (mung bean)^63^ and Sharma et al. (moth bean)^64^, who discovered that a decrease in water potential corresponded with a reduction in the number of roots per plant. Additionally, it was shown that increasing the K supply to promote root development resulted in an increased in the root surface area^65^. Between the genotypes, G1 recorded more advanced root growth than G2 concerning water status (stress and non-stress) and K fertilization. This clearly demonstrates that G1 is WS tolerant and G2 is susceptible.

### Effects of additional K fertilization with RKF on the yield contributing traits and yield of mung bean under WS condition

#### Plant height (cm)

Mung bean genotypes, elevated K levels, and the interplay between genotypes and K levels all had a substantial impact on plant height (Table 1 and Supplementary Table S4)). The mung bean genotype G1 produced taller plants while G2 produced shorter plants in all conditions. WW plants treated with RKF treatment (KL_1_) gave the tallest plant whereas the shortest plant was noted in WS with RKF treatment (KL_2_). The reduction in plant height under drought stress may be attributed to hormonal imbalances, specifically disruptions in cytokinin and abscisic acid levels affecting growth by altering cell wall extensibility, as well as plant dehydration, which might have contributed to decreased relative turgidity leading to turgor loss, decreased cell expansion, and decreased cell division^66^. Increasing K levels (25, 50, 75, 100, and 125%) with KL_2_ (KL_3_–KL_7_) significantly increased the plant height which was 1, 14, 17, 32, and 35%, respectively. However, KL_7_ showed the tallest plant. The increased plant height under greater K levels may be related to the soil's adequate K content, which promoted plant cell division and expansion and significantly increased plant height. The findings align with Das^67^ research, suggesting that K fertilizer treatment may cause a significant rise in plant height by strengthening the stem and enhancing plant vigor. This is further supported by the observation that a lower root shoot ratio associated with reduced K absorption, resulted in minimal plant height in plots without K application, as noted by Rahman et al.^68^ .

**Table 1:** Effects of additional K on the yield contributing traits at harvest of mung bean genotypes under WS (average of 2018 and 2019).

Treatments	Plant height (cm)	Pods plant^−1^ (no.)	Pod length (cm)	Seeds pod^-1^ (no.)
Potassium fertilization levels (KL)				
KL_1_	56.87a	20.52a	8.11a	11.50a
KL_2_	37.07d	9.95f	5.56d	6.72g
KL_3_	37.35d	10.98e	6.27c	7.63f
KL_4_	42.30c	13.28d	6.54bc	8.01e
KL_5_	43.42c	14.72c	6.64bc	8.73d
KL_6_	48.82b	15.45bc	6.92b	9.80c
KL_7_	50.07b	16.13b	7.08b	10.53b
CV (%)	3.96	2.18	9.07	4.45
LSD (0.05)	1.59	0.85	0.54	0.36
Genotypes				
G1	47.54a	15.56a	7.83a	10.25a
G2	42.71b	13.31b	5.63b	7.73b
CV (%)	4.26	5.18	9.28	6.67
LSD (0.05)	0.85	0.33	0.28	0.26
Interactions				
KL × G	***	*	ns	*

KL_1_ = WW + RKF(18 kg K ha^−1^); KL_2_ = WS + RKF(18 kg K ha^−1^); KL_3_ = WS + RKF + 25% additional K; KL_4_ = WS + RKF + 50% additional K; KL_5_ = WS + RKF + 75% additional K; KL_6_ = WS + RKF + 100% additional K; KL_7_ = WS + RKF + 125% additional K; *LS* level of significance, *ns* nonsignificant at P = 0.05.

*Significant at P = 0.05; ***significant at P ≤ 0.001; values within the same column having different letter(s) differed significantly at 5% level of probability as per LSD.

#### Pods plant^−1^

The outcome showed that mung bean genotypes, K levels, and their interaction influenced the pods plant^−1^ (Table 1 and Supplementary Table S4). WS drastically reduced the number of pods plant^−1^ in both the genotypes compared to WW conditions. The KL_1_ treatment gave the highest pods plant^−1^ whereas the minimum pods plant^-1^ was noted in the KL_2_ treatment. Mamun et al.^69^ found higher pods plant^−1^ in soybean by applying K which is corroborative to the current results. In this study, increasing K fertilizer with RKF in WS (KL_3_–KL_7_) significantly increased the pods plant^−1^ by 10, 33, 48, 55, and 62%, respectively. However, the mung bean plants received K 125% (KL_7_) and produced the maximum number of pods plant^−1^. The availability of K in drought-prone soil perhaps promoted the growth and metabolic activity which increased the number of pods plant^−1^. A higher number of pods plant^−1^ was recorded in the G1 genotype than in the G2 genotype (Table 1). It was also observed that under the KL_2_ treatment number of pods, plant^−1^ reduced by 50 and 53% as compared to KL_1_ in G1 and G2 genotypes, respectively. This finding was consistent with mungbean and cowpea by Pandey et al.^70^. The increase in the pods plant^−1^ was mainly associated with increased plant height. Similar results were observed by Tariq et al.^71^ who reported that K application significantly increased the number of pod bearing, branches plant^−1^, and seed yield in mung bean.

#### Pod length (cm)

WS condition (KL_2_) significantly reduced the pod length in both the genotypes compared to WW condition (KL_1_). Rising K fertilizer at the rate of 25, 50, 75, 100, and 125% with RKF under WS conditions (KL_3_–KL_7_) significantly increased the pod length by 13, 18, 19, 24, and 27%, respectively. Mung bean plants that received 125% additional K (KL_7_) produced the longest pod, compared to those subjected to lower K levels. Sufficient K in water-stressed soil contributed to the maintenance of better water status and crop growth which might have enhanced the pod length. In between the genotypic performance, the extended pod length was noted in the G1 genotype and shorter in G2 (Table 1). Previous studies also noted a significant effect of K on pod length^53^.

#### Seeds pod^−1^

Under WS (KL_2_), the seeds pod^−1^ significantly decreased from 11.50 to 6.72; this reduction was recorded as 41.57% when compared to KL_1_ (Table 1). The results of this study showed that K has a significant effect on the number of seeds pod^−1^. Application of additional K (KL_3_–KL_7_)under WS considerably increased the seeds pod^−1^ from 7.63 to 10.53, with the recorded increments of 14, 19, 30, 46, and 57%, respectively. The increase in seeds pod^-1^ was mostly linked to longer pod length and increased plant height, attributed to the greater K supply ^72^. Furthermore, the presence of sufficient K in the root zone helped in maintaining a higher moisture level (Table 1), higher TDM, and crop growth (Figs. 2 and 4) which might be enhanced by longer pod length and number of seeds pod^−1^. The results also suggested that the experimental soil had a medium level of K and the application of higher K doses perhaps increased the seeds pod^−1^. The number of seeds pod^−1^ varied remarkably between the genotypes, and the G1 genotype showed superiority by producing the number of seeds pod^−1^ compared to the genotype G2 which recorded a lower seeds pod^−1^ as shown in Table 1. The interaction between K levels and genotypes also demonstrated significant differences (Supplementary Table S4). The observed difference in the seeds pod^−1^ between genotypes may be attributed to their distinct genetic compositions. According to the results of Jamil et al.^73^, seeds pod^−1^ was found to increase with an increased supply of K.

#### Thousand seed weight (g)

Thousand seed weights (TSW) of respective harvesting time, as well as their average value, were significantly varied by mung bean genotypes, K levels, and their interaction (Table 2 & supplementary Table S4).). The TSW also varied with the picking times as the first picking was followed by the second and the third picking. The treatment KL_2_ (WS) significantly reduced the TSW at all picking times as well as the average TSW in both genotypes. Additional K fertilization (KL_3_–KL_7_)in stress conditions led to an increase in TSW across all the harvesting times and the average TSW. However, KL_7_ resulted in a 13.50% higher increase in TSW over KL_2_. Between the genotypes, G1 consistently gave higher TSW in all the picking times as well as their average TSW data than G2. The higher grain weight in the G1 genotype might be due to variations of genetic makeup as well as enhanced assimilate translocation to the grain during the grain-filling period. Data showed that KL_2_ treatment decreased the TSW by 13 and 19% compared to KL_1_ in G1 and G2, respectively. However, treatment KL_3_–KL_7_ increased the substantial amount of TSW by 2.36, 5.07, 7.21, 8.20, and 9.32% in G1 and 7.27, 13.07, 14.80, 17.85, and 18.44% in G2, respectively. The results revealed that the G2 genotype (stress susceptible) responds more favorably to K fertilizer than the G1 genotype (moderate stress-tolerant) with respect to increased TSW under WS conditions. The study's findings are consistent with past research showing that seeds from plants deficient in K are often tiny, shriveled, and more susceptible to disease, and that seed quality can be improved by applying an appropriate quantity of K^74^. According to Nejat et al.^75^, WS applied to maize during the blooming stage decreased the grain weight by 19%. It is noted that the maturity stage has a substantial impact on grain weight, and WS applied at a later development stage may dramatically decrease TSW^76^.

**Table 2:** Effect of additional K fertilization with RKF on the thousand seed weight (g) of mung bean genotypes under WS (average of 2018 and 2019).

Treatments	1st picking	2nd picking	3rd picking	Average
Potassium fertilization levels (KL)				
KL_1_	37.39a	36.72a	31.98a	35.37a
KL_2_	30.76f	30.30f	28.06d	29.71f.
KL_3_	32.37e	31.47e	29.40c	31.08e
KL_4_	33.90d	33.38d	29.63bc	32.30d
KL_5_	34.48c	34.24c	29.93bc	32.88c
KL_6_	35.30b	34.98b	30.08bc	33.45b
KL_7_	35.55b	35.19b	30.41b	33.72b
CV (%)	1.34	2.20	3.13	1.47
LSD (0.05)	0.41	0.66	0.83	0.43
Mung bean genotypes				
G1	36.28a	35.74a	31.03a	34.35a
G2	32.21b	31.76b	28.82b	30.93b
CV (%)	1.84	3.51	2.99	2.41
LSD (0.05)	0.28	0.52	0.39	0.35
Interactions				
KL × G	*	*	*	*

KL_1_ = WW + RKF (18 kg K ha^-1^); KL_2_ = WS + RKF (18 kg K ha^-1^); KL_3_ = WS + RKF + 25% additional K; KL_4_ = WS + RKF + 50% additional K; KL_5_ = WS + RKF + 75% additional K; KL_6_ = WS + RKF + 100% additional K; KL_7_ = WS + RKF + 125% additional K; *LS* level of significance.

*Significant at P ≤ 0.05; RKF = RKF; values within the same column having different letter(s) differed significantly at 5% level of probability as per LSD.

#### Seed yield

Seed yield significantly varied by mung bean genotypes, increasing K levels and their interaction (Table 3 and supplementary Table S5). At WW treatment (KL_1_) the maximum seed yield was found in the third picking followed by the first picking and the second picking. But in the first picking, the highest seed yield was gained in the rest of the treatment (KL_2_–KL_7_) followed by the third picking and the second picking. In the case of all the harvesting times and average yield, increased K fertilizer with RKF in stress conditions increased the seed yield. However, higher average yield was obtained from increasing K levels (KL_3_–KL_7_) as compared to additional K non-fertilized plots (KL_2_). The mung bean yield exhibited a notable increase of 32.52% when receiving 125% more K (KL_7_) compared to the application of RKF alone (KL_2_) under WS conditions. Higher seed yield was obtained from higher K levels due to maintaining better water relation, plant growth, LAI, TDM increasing plant height, pods plant^−1^ as well as lengthened pod development.

**Table 3:** Effect of additional K fertilization with RKF on the seed yield (kg ha^−1^) of mung bean genotypes under WS (average of 2018 and 2019).

Treatments	1st picking	2nd picking	3rd picking	Average
Potassium fertilization levels (KL)				
KL_1_	458.72a	410.44a	541.22a	1410.37a
KL_2_	317.39c	240.91d	266.87d	825.17e
KL_3_	381.03b	279.46bc	284.75d	945.24d
KL_4_	385.99b	264.93cd	337.33c	988.24d
KL_5_	385.36b	285.17bc	329.55c	1000.08cd
KL_6_	390.44b	286.84bc	380.09b	1057.37bc
KL_7_	409.90b	300.90b	382.69b	1093.50b
CV (%)	9.39	11.17	10.21	6.48
LSD (0.05)	32.55	29.37	32.72	60.23
Genotypes				
G1	264.52a	402.44a	458.08a	1125.04a
G2	515.14b	188.60b	262.64b	966.38b
CV (%)	5.66	7.28	5.81	4.45
LSD (0.05)	9.72	9.48	9.23	20.47
Interactions				
KL × G	***	***	***	***

KL_1_ = WW + RKF(18 kg K ha^−1^); KL_2_ = WS + RKF (18 kg K ha^−1^); KL_3_ = WS + RKF + 25% additional K; KL_4_ = WS + RKF + 50% additional K; KL_5_ = WS + RKF + 75% additional K; KL_6_ = WS + RKF + 100% additional K; KL_7_ = WS + RKF + 125% additional K; *LS* level of significance.

***significant at P ≤ 0.001; Values within the same column having different letter(s) differed significantly at 5% level of probability as per LSD.

Between the genotypic means, G2 achieved the highest seed yield in the first picking followed by the third picking and the second picking. In contrast, the highest seed yield for G1 genotype was observed in the third picking, following the second and the first picking. However, overall higher seed yield was observed in G1, which exhibited a 16% greater seed yield compared to G2. Fertilization of K at rates of 25, 50, 75, 100, and 125% more (KL_3_–KL_7_) with RKF under WS (KL_2_) positively influenced the seed yield of both genotypes and is also noted that the seed yield increased by 3.55, 8.72, 9.52, and 17.08, 23.30% in G1 and 29.88, 35.15, 37.48, 43.56, 45.37% in G2, respectively. The results showed that the genotype G2 exhibited a better K response with respect to seed yield increment under WS conditions than the G1 genotype. The increases in plant height, pods plant^−1^, and pod length were mostly linked to an increase in seed output.

According to earlier research, crop yield and quality are adversely affected by the low level of K in the soils^77^. However, the impact of K on both the quantity and quality of beans was investigated, revealing a significant influence on grain output by positively affecting the number of pods and grains per pod^78^. Furthermore, K treatment has been shown to enhance the photosynthetic rate and increase the availability of other soil nutrients, which raised seed yield^79^.

#### Correlation analysis

**Figure 6:**
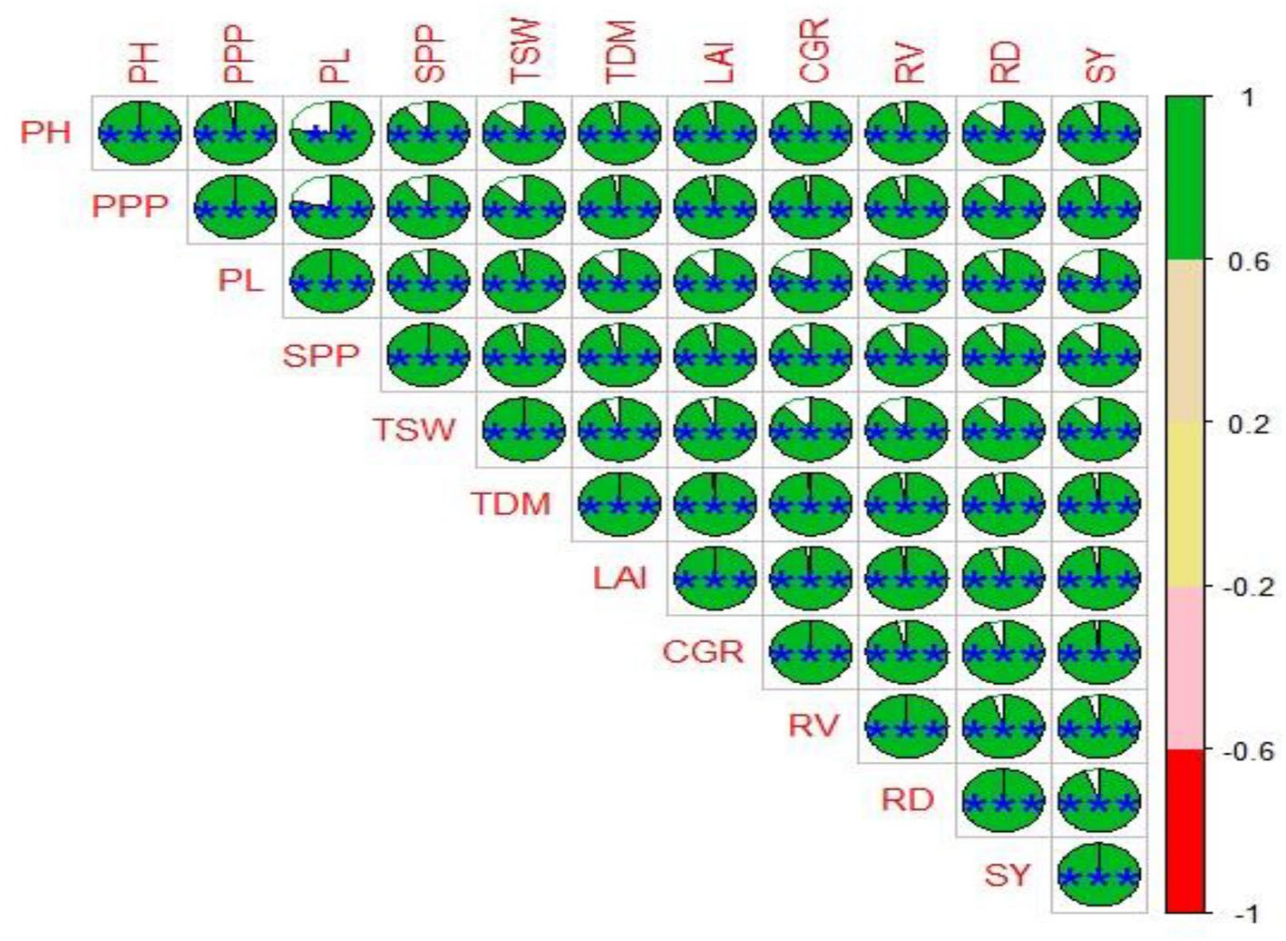
Correlation coefficients of yield contributing and growth traits with seed yield of mung bean genotypes under different treatments. The deeper positive color scale signifies most of the treatment reactions, whereas deeper negative stripes display less response. The deep green color presented a highly positive relationship and the deep red color exposed a high negative association between traits. Likewise, decreasing the color intensity, reducing the performance of the treatment in both the positive and negative ranges (***significant at P ≤ 0.001; *PH* plant height, *PPP* pods plant^−1^, *PL* pod length, *SPP* = seeds pod-1, *TSW* 1000-seed weight, *TDM* total dry matter (at harvest), *LAI* leaf area index (55 DAS), *CGR* crop growth rate (55 DAS), *RV* root volume, *RD* root density,* SY* seed yield.

A common method for assessing the strength of the association between two or more variables is the correlation matrix. Choosing a program based on the correlation matrix may be an excellent way to test for better genotypes^80^. The correlation analysis results showed that almost every statistic had a significant link with every other parameter (Fig. 6). For every variable under study, there was a strong positive association between seed yield and each attribute. From this correlation study, the maximum significant positive correlation (˃ 0.90) was recorded within the seed yield with RV (0.94), RD (0.92), and pods plant^−1^ (0.92); RD with RV(0.93); RV with pods plant^−1^ and thousand seed weight with pod length and seeds pod^−1^ (0.94), and plant height with pods plant^−1^ (0.93). These results are in agreement with earlier findings ^81,82^.

### Effects of applied additional K fertilizer with RKF on nutrient content in mung bean stover and seeds under WS condition

The nutrient content in mung bean stover and seeds was found significantly difference among the K levels and genotypes (Table 4) but their interaction was observed with no significant variation (supplementary Table S6**).**

**Table 4:** Effects of additional K fertilization on the nutrient content in mung bean stover and seed (average of 2018 and 2019).

Treatments	Stover				Seed			
	N (%)	P (%)	K (%)	S (%)	N (%)	P (%)	K (%)	S (%)
Potassium fertilization levels (KL)								
KL_1_	0.91a	0.17a	1.60a	0.21a	8.71a	0.23a	2.01a	0.55a
KL_2_	0.66f	0.15d	0.93e	0.13e	6.18f	0.19b	1.48d	0.40d
KL_3_	0.69ef	0.15d	0.96e	0.13e	6.68e	0.20b	1.50d	0.43cd
KL_4_	0.73de	0.15d	1.10d	0.15d	7.00de	0.21b	1.64cd	0.47bc
KL_5_	0.75cd	0.16bc	1.13d	0.17c	7.18d	0.21b	1.68cd	0.52ab
KL_6_	0.79c	0.16bc	1.26c	0.18bc	7.65c	0.23a	1.80bc	0.54a
KL_7_	0.84b	0.17a	1.39b	0.19b	8.10b	0.24a	1.94ab	0.56a
CV (%)	5.85	7.48	6.64	12.58	6.02	8.00	13.14	12.68
LSD (0.05)	0.04	0.011	0.071	0.019	0.39	0.015	0.20	0.056
Genotypes (G)								
G1	0.82a	0.16a	1.26a	0.17a	7.85a	0.23a	1.80a	0.51a
G2	0.71b	0.15b	1.13b	0.16b	6.86b	0.20 b	1.64b	0.48b
CV (%)	7.41	10.64	11.03	14.84	8.96	16.87	11.94	11.14
LSD (0.05)	0.02	0.007	0.058	0.011	0.29	0.016	0.09	0.024
Interactions								
KL × G	ns	ns	ns	ns	ns	ns	ns	ns

KL_1_ = WW + RKF (18 kg K ha^−1^); KL_2_ = WS + RKF; KL_3_ = WS + RKF + 25% additional K; KL_4_ = WS + RKF + 50% additional K; KL_5_ = WS + RKF + 75% additional K; KL_6_ = WS + RKF + 100% additional K; KL_7_ = WS + RKF + 125% additional K; *LS* level of significance, *ns* nonsignificant.

*Significant at P ≤ 0.05; **significant at P ≤ 0.01; ***significant at P ≤ 0.001_;_ Values within the same column having different letter(s) differed significantly at 5% level of probability as per LSD.

#### Nitrogen content

Under water stress (WS) conditions, N uptake by mung bean genotypes decreased significantly (Table 4). In the present study, increasing K levels under WS, particularly up to 125% with RKF doses, gradually raised N content in both mung bean stover and seed. The substantial increase in N content in both stover and seeds accounting for 27.27% and 30.07% respectively, was observed with KL_7_, followed by the application of 100% additional K (KL_6_), which resulted in increases of 19.70% and 23.79% respectively. On the other hand, N content in stover and seed under KL_1_ increased by 37.88 and 40.94% as compared to KL_2_.

A comparison between genotypes revealed that G1 outperformed G2 in N uptake. G1 exhibited higher N content (0.96 and 0.71%; 9.11 and 6.63%) in stover and seeds compared to G2 (0.86 and 0.60%; 8.30 and 5.75%) under both KL_1_ and KL_2_ treatments, respectively. The highest N content was recorded in the genotype G1 than in G2 under both conditions and other treatments. Despite both genotypes showing reduced N content under KL_2_ compared to KL_1_, G1 experienced a lower reduction (26.04% in stover and 27.22% in seeds) compared to G2 (30.23% in stover and 30.84% in seeds).

Water stress is known to cause a rapid buildup of free amino acids, leading to higher N content in plants ^83^. The extended existence of water stress in soil hampers the movement of N, leading to a deficit in N availability ^84^, hampers plant development, and can cause chlorosis in peas^85^. Moreover, WS may lessen soil-N mineralization, which would limit N availability^86^. A slower rate of transpiration affecting the transfer of N from roots to shoots might potentially be the cause of a reduced N intake crop^87^. The application of K fertilizer under water stress has been shown to improve plant water relations, facilitating increased N absorption from soil and subsequently higher N levels in plant tissue^88^.

#### Phosphorus content

The current study showed significant differences in P content in mung bean stover and seeds with additional K fertilization (Table 4). The highest P content (0.17% in stover and 0.23% in seeds) was observed under KL_1_ treatment, while the lowest (0.15% in stover and 0.19% in seeds) was recorded in KL_2_. Additional K application alongside RKF improved P uptake under WS (KL_3_–KL_7_), with the highest P content (0.17% in stover and 0.24% in seeds) achieved with 125% additional K (KL_7_), followed by 100% additional K (KL_6_), representing increases of 13.33% to 26.32% compared to KL_2_ treatment.

The genotype G1 exhibited higher P content compared to G2, which might be due to the genetic makeup and inherent capacity. WS negatively affected P uptake regardless of genotypes, with G2 showing a lower uptake under RKF and WS conditions (KL_2_). The increase in K levels (25–125% with RKF; KL_3_–KL_7_) positively influenced P content in both stover and seeds under WS conditions which ranged from 0.15 to 0.17% in stover and 0.21 to 0.25% in seed for G1 and 0.15 to 0.16% in stover and 0.19 to 0.23% in seed for G2. It was noticed that WS decreased Ca, Mg, and P concentrations in wheat plants^89^. The P contents significantly reduced under WS conditions in rice genotypes and the reduction was lesser in tolerant genotypes than in susceptible ones^90^. Previous research indicates that WS reduces P absorption and transport in plants^91,92^, resulting in decreased P concentrations in various crops, primarily because of little increase in aerial biomass^93^.

#### Potassium content

Potassium plays a vital role in plant physiology, acting as an osmotic regulator^94^ and balancing nutrient effects of both N and P and thus, it is especially important in multi-nutrient fertilizer applications^95^. The K fertilizer levels and varying variable moisture regimes significantly influenced the K content in mung beans (stover and seed) (Table 4). The treatment KL_1_ achieved the maximum K content in stover and seeds (1.60 and 2.01%) which was 72.04 and 35.81% more as compared to KL_2_ treatment (0.93 and 1.48%). The K content in mung bean (stover and seed) was significantly reduced by the WS plot (KL_2_). While, the application of additional K fertilizer at the rate of 25, 50, 75, 100, and 125% (KL_3_-KL_7_) with KL_2_ led to an increased the K content in mung bean stover and seeds which ranged from 0.96 to 1.39% and 1.50–1.94%, respectively.

Between the mung bean genotypes, G1 was statistically more capable of gaining more K content compared to G2 under both WW (KL_1_) and WS conditions (KL_2_). However, under KL_1_, the K content in both stover and seed was statistically similar between the genotypes, with negligible variation (1.63 and 1.57%; 2.15 and 1.88%, respectively). The genotype G2 experienced a significant decrease in K content, while G1 showed an increase compared to the control. WS reduced K uptake by plants, but the application of additional K fertilizer under WS conditions increased K uptake. Tolerant genotypes generally show higher K values^96^.WS reduced K concentration in wheat genotypes but the tolerant ones were less affected as compared to WW ones^97^. Plants required more internal K to recover from drought stress^98^, and K played a key role in the osmotic adjustment including stomatal opening contributing to improved yield upon K application to plants^99^. Application of K to soil significantly influenced N, P, K, protein, and Fe in faba bean^100^, promoting nutritional balance, and enhancing photosynthate production and mineral element uptake ^101,102^.

#### Sulphur content

The availability of S depends on soil moisture as reported by Itanna ^103^. However, under WS condition K increases the plant and soil water status by improving the root architecture which enhances the water flow with different nutrient elements from soil to plant parts. From this study, it was observed that the S content in mung bean stover and seeds significantly varied with the variation in water regimes as well as increasing K level (Table 4). The S content in mung bean stover and seeds showed 0.21 and 0.13%; and 0.55 and 0.40% under KL_1_ and KL_2_ treatment, respectively. However, the addition of K at the rate of 25, 50, 75, 100, and 125% with RKF under WS conditions (KL_2_) increased the stover and seeds' K content.

Regarding mung bean genotypes, G1 exhibited a higher S content compared to G2 under both WW (KL_1_) and WS (KL_2_) conditions with RKF. However, under KL_1_, the stover and seed S content were statistically identical between the genotypes. Additionally, the G1 genotype showed a lower S content than G2 under WS compared to WW conditions. WS can induce modifications in the metabolism of amino acids and proteins ^104^. It is plausible that S-deficient plants may experience these phenomena in response to WS.

## Materials and methods

### Site and test crop

A field experiment was executed under a rainout shelter at Regional Research Station, Ishwardi, Pabna, Bangladesh during two sequential summer seasons of 2018 and 2019 to determine the effect of additional K fertilization in relieving the adverse effect of drought in response to WUE, growth, nutrient content, yield attributes and yield of summer mung bean. The location was at 24.03° North latitude, 89.05° East longitude, and 16 m altitude above the sea level under ‘High Ganges River Floodplain soil’ (Agro-ecological Zone-11) in Bangladesh. Mung bean genotypes viz., BMX-08010–2 (WS-tolerant: G1) and BARI Mung-1 (WS-sensitive: G2) were used in this experiment ^105^. BARI Mung-1 and BMX-08010–2 have released varieties and advanced breeding lines, respectively developed from Pulses Research Centre and Regional Agricultural Research Station, Bangladesh Agricultural Research Institute (BARI), Ishwardi, Pabna, Bangladesh. Experimental research and field studies on mung bean including the collection of mung bean seeds comply with our institutional, national, and international guidelines and legislation.

### Soil and agro-climatic condition

**Figure 7:**
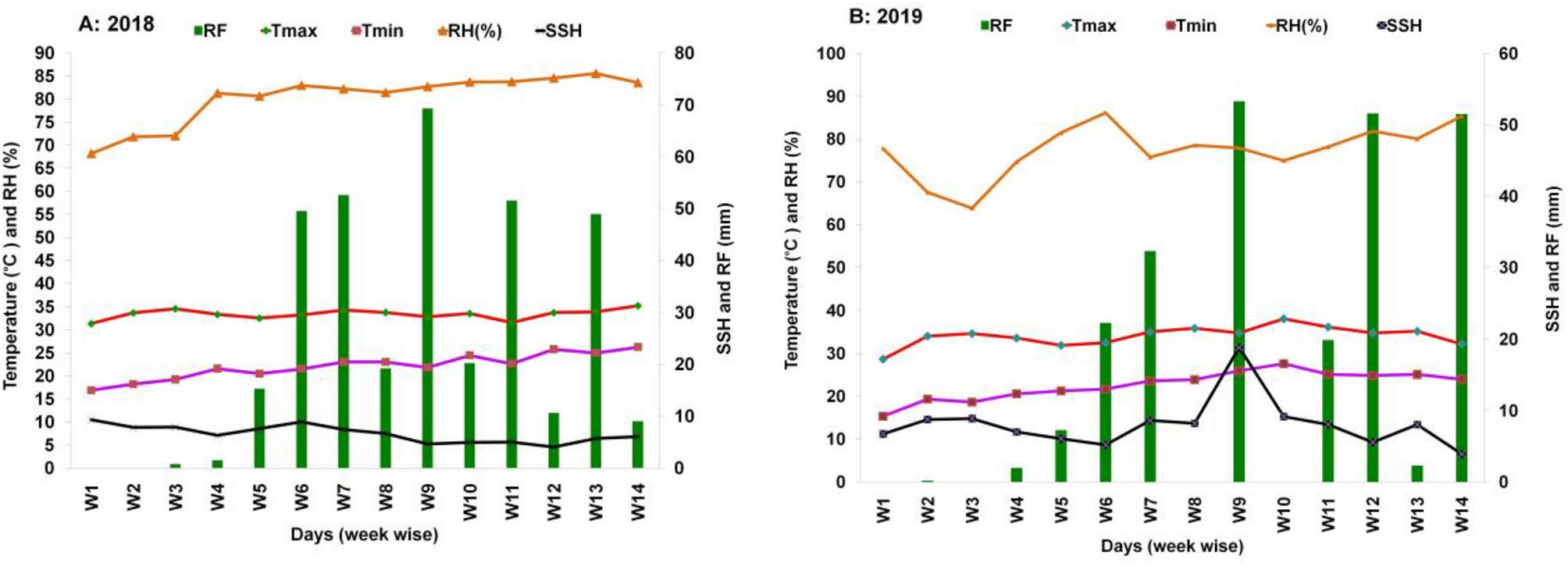
Weekly average temperature, relative humidity (RH), sunshine hour (SSH; day^−1^), and total rainfall (RF: mm) prevailed (4th March–8th June) during the two successive growing periods of 2018 and 2019 (*Tmax* maximum temperature, *Tmin* minimum temperature, *W1* first week to *W14* 14th week).

The physical and chemical properties of the soil were analyzed before mung bean sowing from the depth of 0–15 cm soil profile. The field capacity, bulk density, and permanent wilting point of the soil, which was clay loam in texture and slightly alkaline in nature, were 29.20%, 1.40 g/cc, and 13%, respectively. The soil contained organic matter (1.23%), pH (7.30), N (600 mg kg^−1^), P (34.01 mg kg^−1^), K (0.25 cmol kg^−1^), S (42.43 mg kg^−1^), boron (0.29 mg kg^−1^), zinc (0.60 mg kg^−1^). The meteorological data, which was collected from Bangladesh Sugar Crops Research Institute situated about 400 m from the experimental site, with respect to temperature, rainfall, and relative humidity during the growing period of the experimental site are presented in Fig. 7.

### Experimental design and treatments

The experiment was conducted in a split-plot design with three replications. Two mung bean genotypes were placed in the main plots as Factor A: BMX-08010-2 (WS tolerant: G1) and BARI Mung-1 (WS susceptible: G2), whereas seven K fertilization treatments were placed randomly in the sub-plots as Factor B: (1) well-watered (WW) + recommended K fertilization (RKF i.e. 18 kg K ha^−1^) (KL_1_), (2) WS + RKF (KL_2_), (3) WS + 25% additional K with RKF (KL_3_), (4) WS + 50% additional K with RKF (KL_4_), (5) WS + 75% additional K with RKF (KL_5_), (6) WS + 100% additional K with RKF (KL_6_), (7) WS + 125% additional K with RKF (KL_7_) maintaining 3 m × 3 m plots size. The RKF used was determined by Azad et al.^106^.

### Fertilization

The blanket dose of N–P–S–B fertilizers was applied at the rate of 20–17–10–2 kg ha^−1^ in the form of urea, triple superphosphate, gypsum, and boric acid, respectively^106^. Potassium (K) fertilizer was incorporated in the soil as per treatments in the form of muriate of potash (KCl), while the entire amount of fertilizers was added to the soil during the final land preparation (three days before sowing).

### Seed sowing and crop management

Mung bean seeds were sown in continuous seeding maintaining 30 cm line apart rows on 4 March 2018 and 2019, respectively. The seed rate was used at the rate of 25 kg ha^−1^. The seed was treated before sowing with Carboxin and Thiram combination (Provax-200 WP) maintaining 3.5 g per kg seeds for controlling seed & soil-borne diseases. After 15 days of emergence, the plants were thinned by maintaining a distance of 6–7 cm followed by hand weeding. Subsequently, weed control was done 30 days after sowing (DAS). Imidachloprid (Imitaf 20 SL) at the rate of 0.5 mL L^−1^ of water was used for controlling flower thrips spraying at 35 DAS and 45 DAS. After 45 DAS, Lambda-Cyhalothrin (Karate 2.5 EC/Reeva 2.5 EC) at the rate of 1 mL L^−1^ of water was applied 3–4 times at seven days intervals for controlling of pod borer.

### Maintenance of soil moisture

Following seed planting in each treatment, general irrigation (53 mm) was applied to ensure optimal germination and seedling establishment depending on the moisture content of the soil. Following that, three irrigations were given to WW treatments only^107^ at DAS 20, 35, and 45. Taking into account the 45 cm effective root zone depth, irrigation was delivered up to the field capacity levels based on soil moisture content at each irrigation time^108^. The estimated volume of water was manually administered in a specific flood irrigation treatment. In contrast, the crop in the water-stressed treatment was protected from rains by a rainout shelter, and the WS condition was sustained without irrigation for the duration of the growth season. The net quantity of irrigation water formula is shown below.

$${\text{Net amount of irrigation water = }}\frac{{\text{FC - MC }}}{{{100}}} \times p \times D$$

where, FC is the field capacity of the soil (%); MC is the moisture content of the soil at the time of irrigation (%); p is the bulk density of the soil (g/cc), and D is the root zone depth (cm).

The soil water depletion (SWD) in the effective root zone depth was estimated according to the below mentioned formula^109^:

$$SWD = \sum\limits_{i = 1}^{n} {} \frac{{\text{(Mbi - Mei)}}}{{{100}}} \times BDi \times Di$$

whereas SWD is the depletion of soil water (mm); Mbi is the percentage of moisture in a particular soil layer at the start of the season; Mei is the percentage of moisture in a certain soil layer after the growing season; n is the number of soil layers in the root zone (3); BDi stands for bulk density of a particular soil layer, and Di for depth (mm) of a particular soil layer within the root zone.

The exploited irrigation water by the crop was measured according to the following formula^110^:

$${\text{Water use efficiency (WUE) }} = \frac{{{\text{Yield (kg ha}}^{{{ - }{1}}} {)}}}{{\text{Total water use (mm)}}}$$

### Measurement of growth and yield traits

Ten plant samples were objectively taken at various phases of development (20 DAS-first trifoliate leaf stage; 25 DAS-3rd trifoliate leaf stage; 35 DAS-flower initiation stage; 45 DAS-pod filling stage; 55 DAS-pod color change stage and harvesting stage-when pod become black color i.e., harvesting maturity) to measure the CGR^111^, LAI^112^, and dry matter accumulation. At the final harvesting stage, root sample was conducted in each repeated plot per treatment to determine the root volume and root density. In this instance, the roots were extracted using a root sample instrument, and the volume was still 4500 cm^3^ (height 20 cm × top 15 cm × base 15 cm). After sampling, the roots were carefully washed under flowing tap water. A 100 ml measuring cylinder was used to assist in calculating the root volume volumetrically. First, 50 ml of tap water were added to the cylinder, and all of the roots were submerged in the liquid. It was noted that the roots' soaking had raised the water level. Root volume was defined as the difference between the final and beginning volumes. After the samples were oven-dried for 72 h 80 °C, the root dry weight was measured, and the root density was ascertained.

Indiscriminately ten plants were tagged earlier for measuring the yield contributing traits of particular treatment plots. The crop was harvested from an area of 2.0 m × 1.5 m (3 m^2^) from the undisturbed middle area of the unit plots. Mung bean pods were harvested three times at 60–65, 70–75, and 90–95 DAS, respectively when they had turned blackish-brown in color, and dried to such a level that they were about to shatter. The threshed seeds were dried, cleaned, and weighed, and finally maintained the moisture content of the grain (~ 10%) was through frequently observing by a moisture meter (model F/RMEX).

$${\text{Crop growth rate }}\left( {{\text{CGR}}} \right) \, = \frac{{{\text{(TDM)t}}_{{2}} \, { - }{\text{ (TDM)t}}_{{1}} }}{{{\text{t}}_{{2}} \, { - }{\text{ t}}_{{1}} }} {\text{g plant}}^{{ - {1}}} {\text{day}}^{{ - {1}}}$$

where, TDM = total dry matter, t_1_ = time of first observation, t_2_ = time of second observation

$${\text{LAI }} = \frac{{\text{Leaf area/plant}}}{{\text{Ground surface area/plant}}}$$

### Estimation of N, P, K, and S content of stover and seeds of mung bean

The Macro-Kjeldhal method^113^ was used for measuring the N content in the stover and seeds of mung bean crops of different treatments after digestion with concentrated H_2_SO_4_, H_2_O_2,_ and digestion tablet (catalyst mixture) which was prepared by mixing K_2_SO_4_, CuSO_4_.5H_2_O, and selenium powder in the ratio of 100:10:1, respectively. Then N in the digest was measured by distillation with 40% NaOH followed by titration of the distillate trapped in H_3_BO_4_ with 0.01 N H_2_SO_4_. Nitrogen content in each sample was predicted with the help of a standard curve, which is expressed as a percentage.

The analysis of elements P, K, and S was done according to Jones and Case^114^, which is mentioned below-

Phosphorus content in the digested sample was determined by adding freshly prepared SnCl_2_ solution to develop the blue color of the phosphomolybdate complex, and the color was measured by spectrophotometer at 660 nm of incident light followed by calibration of the reading with a standard curve of P. The K content was determined directly from the extract by using a flame photometer. Sulphur **(**S) content in the digest of mung bean samples was determined by adding 5 mL distilled water, 1 mL acid seed solution, and 0.5 g BaCl_2_ crystal and measuring the turbidity with the help of a spectrophotometer at 420 nm.

### Statistical analysis

The same trend of results among the studied traits was found between the two successive years. So, pooled analysis was done from the obtained values of the respective traits. In this case, the measured data were analyzed statistically using computer-based R-stat software (version 3.1.2) following the basic procedure given by Gomez and Gomez^115^. The treatments' significant level was ascertained by analysis of variance (ANOVA), which was then contrasted using the Least Significant Difference Test (LSD). To investigate the link between the desired variables, correlation analysis was performed.

In conclusion, the results exhibited that the application of additional K fertilizer with the recommended dose under WS conditions is considered the most suitable for realizing the highest safe yield of mung beans and it can alleviate the harmful effects of WS through improved water productivity, growth, yield contributing traits and NPS accumulation performance. The genotype G1 was more resistant to WS compared to G2 for giving a higher response to all the measured traits and yield. Finally, the findings lead to recommend that mung bean crops grown under WS require additional K to minimize the negative effect of WS and help to accomplish sustainable productivity and quality of mung bean crops.

### Supplementary Information

Supplementary Information.

Supplementary Tables.

## Data Availability

All data generated or analyzed during this study are included in this published article and its supplementary information files.
