# Supplementary material for: Potassium augments growth, yield, nutrient content, and drought tolerance in mung bean (Vigna radiata L. Wilczek.)
Source: Sci Rep. 2024 Apr 23;14:9378. doi: 10.1038/s41598-024-60129-z (PMC11039697; doi:10.1038/s41598-024-60129-z)
Supplement: Supplementary file 2 — Supplementary Tables. [file 41598_2024_60129_MOESM2_ESM.docx]

**Supplementary Table S1. Combined effects of additional K fertilization and mungbean genotypes on TDM (average of 2018 and 2019)**

| Interactions | TDM (g) | | | | | |
| --- | --- | --- | --- | --- | --- | --- |
|  | 20 DAS | 25 DAS | 35 DAS | 45 DAS | 55 DAS | Harvest |
| KL_1_ G1 | 0.068 | 0.502 | 1.997 | 6.303 | 12.303 | 13.974 |
| KL_2_ G1 | 0.073 | 0.347 | 1.492 | 3.781 | 6.877 | 11.419 |
| KL_3_ G1 | 0.079 | 0.368 | 1.571 | 3.984 | 7.193 | 10.770 |
| KL_4_ G1 | 0.079 | 0.398 | 1.651 | 4.197 | 8.038 | 10.268 |
| KL_5_ G1 | 0.085 | 0.426 | 1.703 | 4.482 | 8.518 | 9.711 |
| KL_6_ G1 | 0.085 | 0.432 | 1.757 | 4.829 | 9.056 | 9.134 |
| KL_7_ G1 | 0.089 | 0.466 | 1.782 | 4.971 | 9.437 | 8.684 |
| KL_1_ G2 | 0.078 | 0.452 | 1.862 | 4.780 | 10.057 | 8.069 |
| KL_2_ G2 | 0.077 | 0.322 | 1.210 | 2.093 | 4.213 | 7.863 |
| KL_3_ G2 | 0.081 | 0.333 | 1.304 | 2.766 | 5.531 | 7.378 |
| KL_4_ G2 | 0.085 | 0.345 | 1.350 | 3.227 | 6.051 | 7.351 |
| KL_5_ G2 | 0.088 | 0.373 | 1.599 | 3.300 | 6.575 | 6.785 |
| KL_6_ G2 | 0.090 | 0.381 | 1.629 | 3.573 | 6.967 | 6.002 |
| KL_7_ G2 | 0.094 | 0.409 | 1.707 | 3.897 | 7.475 | 4.641 |
| CV (%) | 11.52 | 8.47 | 12.29 | 7.48 | 7.65 | 8.66 |
| LSD (0.05) | 0.01 | 0.04 | 0.23 | 0.35 | 0.69 | 0.88 |
| LS | ns | ns | ns | ns | ns | ns |

KL_1_ = WW + RKF (18 kg K ha^-1^); KL_2_ = WS + RKF; KL_3_ = WS + RKF + 25% additional K; KL_4_ = WS + RKF + 50% additional K; KL_5_ = WS + RKF + 75% additional K; KL_6_ = WS + RKF + 100% additional K; KL_7_ = WS + RKF + 125% additional K; G1= BMX-08010-2; G2= BARI Mung-1; LS = level of significance; ns = nonsignificant

**Supplementary Table S2. Combined effects of additional K fertilization and mungbean genotypes on LAI (average of 2018 and 2019)**

| Interactions | **LAI** (cm^2^ cm^-2^) | | | | | |
| --- | --- | --- | --- | --- | --- | --- |
|  | 20 DAS | 25 DAS | 35 DAS | 45 DAS | 55 DAS | Harvest |
| KL_1_ G1 | 0.016 | 0.120 | 0.433 | 1.219 | 1.377 | 1.122 |
| KL_2_ G1 | 0.017 | 0.081 | 0.357 | 0.722 | 0.787 | 0.592 |
| KL_3_ G1 | 0.019 | 0.083 | 0.369 | 0.757 | 0.829 | 0.655 |
| KL_4_ G1 | 0.018 | 0.091 | 0.388 | 0.798 | 0.893 | 0.673 |
| KL_5_ G1 | 0.020 | 0.099 | 0.390 | 0.846 | 0.942 | 0.752 |
| KL_6_ G1 | 0.020 | 0.100 | 0.400 | 0.929 | 1.002 | 0.872 |
| KL_7_ G1 | 0.021 | 0.110 | 0.406 | 0.949 | 1.044 | 0.946 |
| KL_1_ G2 | 0.018 | 0.107 | 0.402 | 0.865 | 1.119 | 0.796 |
| KL_2_ G2 | 0.017 | 0.077 | 0.290 | 0.350 | 0.470 | 0.279 |
| KL_3_ G2 | 0.019 | 0.079 | 0.294 | 0.451 | 0.626 | 0.442 |
| KL_4_ G2 | 0.019 | 0.079 | 0.295 | 0.537 | 0.652 | 0.474 |
| KL_5_ G2 | 0.020 | 0.089 | 0.369 | 0.543 | 0.750 | 0.513 |
| KL_6_ G2 | 0.020 | 0.089 | 0.371 | 0.600 | 0.840 | 0.638 |
| KL_7_ G2 | 0.021 | 0.097 | 0.392 | 0.656 | 0.892 | 0.707 |
| CV (%) | 11.09 | 30.11 | 10.37 | 14.75 | 16.28 | 11.46 |
| LSD (0.05) | 0.002 | 0.03 | 0.04 | 0.13 | 0.17 | 0.09 |
| LS | ns | ns | ns | ns | ns | ns |

KL_1_ = WW + RKF (18 kg K ha^-1^); KL_2_ = WS + RKF; KL_3_ = WS + RKF + 25% additional K; KL_4_ = WS + RKF + 50% additional K; KL_5_ = WS + RKF + 75% additional K; KL_6_ = WS + RKF + 100% additional K; KL_7_ = WS + RKF + 125% additional K; G1= BMX-08010-2; G2= BARI Mung-1; LS = level of significance; ns = nonsignificant

**Supplementary Table S3. Combined effects of additional K fertilization and mungbean genotypes on CGR (average of 2018 and 2019)**

| Interactions | CGR (g plant^-1^ day^-1^) | | | | | Root volume (cc) | Root density (mg cm^-3^) |
| --- | --- | --- | --- | --- | --- | --- | --- |
|  | 0-20 DAS | 20-25 DAS | 25-35 DAS | 35-45 DAS | 45-55 DAS |  |  |
| G1KL_1_ | 0.0034 | 0.129 | 0.430 | 0.600 | 0.067a | 6.067 | 0.360 |
| G1KL_2_ | 0.0037 | 0.095 | 0.229 | 0.310 | 0.020e | 3.167 | 0.186 |
| G1KL_3_ | 0.0040 | 0.100 | 0.241 | 0.321 | 0.027d | 3.200 | 0.227 |
| G1KL_4_ | 0.0040 | 0.105 | 0.255 | 0.384 | 0.044c | 3.200 | 0.228 |
| G1KL_5_ | 0.0042 | 0.108 | 0.278 | 0.404 | 0.048bc | 3.567 | 0.244 |
| G1KL_6_ | 0.0042 | 0.112 | 0.307 | 0.423 | 0.048bc | 4.167 | 0.267 |
| G1KL_7_ | 0.0045 | 0.113 | 0.319 | 0.447 | 0.053b | 4.367 | 0.275 |
| G2 KL_1_ | 0.0039 | 0.119 | 0.292 | 0.528 | 0.054b | 4.433 | 0.293 |
| G2 KL_2_ | 0.0039 | 0.075 | 0.088 | 0.212 | 0.017e | 2.233 | 0.153 |
| G2 KL_3_ | 0.0041 | 0.081 | 0.146 | 0.276 | 0.019e | 2.300 | 0.157 |
| G2 KL_4_ | 0.0042 | 0.084 | 0.188 | 0.283 | 0.029d | 2.467 | 0.161 |
| G2 KL_5_ | 0.0044 | 0.101 | 0.170 | 0.328 | 0.031d | 2.967 | 0.166 |
| G2 KL_6_ | 0.0045 | 0.103 | 0.194 | 0.339 | 0.044c | 3.333 | 0.182 |
| G2 KL_7_ | 0.0047 | 0.108 | 0.219 | 0.358 | 0.048bc | 3.467 | 0.191 |
| CV (%) | 11.22 | 17.52 | 14.80 | 10.58 | 14.03 | 15.96 | 14.26 |
| LSD (0.05) | 0.001 | 0.02 | 0.04 | 0.05 | 0.01 | 0.65 | 0.04 |
| LS | ns | ns | ns | ns | * | ns | ns |

KL_1_ = WW + RKF (18 kg K ha^-1^); KL_2_ = WS + RKF; KL_3_ = WS + RKF + 25% additional K; KL_4_ = WS + RKF + 50% additional K; KL_5_ = WS + RKF + 75% additional K; KL_6_ = WS + RKF + 100% additional K; KL_7_ = WS + RKF + 125% additional K; G1= BMX-08010-2; G2= BARI Mung-1; LS = level of significance; ns = nonsignificant

**Supplementary Table S4. Combined effects of additional K fertilization and mungbean genotypes on the yield contributing traits and thousand seed weight (average of 2018 and 2019)**

| Interactions | Yield contributing traits | | | | Thousand seed weight (g) | | | |
| --- | --- | --- | --- | --- | --- | --- | --- | --- |
|  | Plant height (cm) | Pods plant^-1^ (no.) | Pod length (cm) | Seeds pod^-1^ (no.) | 1^st^ picking | 2^nd^ picking | 3^rd^ picking | Average |
| G1KL_1_ | 61.20 a | 22.27 a | 9.34 | 12.40 a | 39.12 a | 38.48 a | 33.50 a | 37.03a |
| G1KL_2_ | 40.40 g | 11.10 i | 6.61 | 8.07 h | 33.30 f | 33.27 fg | 29.98c-f | 32.18e |
| G1KL_3_ | 40.77 g | 12.27 h | 7.39 | 8.90 fg | 34.72 e | 33.88 def | 30.23 cde | 32.94de |
| G1KL_4_ | 43.07 ef | 14.37 fg | 7.65 | 9.29 ef | 35.77 d | 35.03 cd | 30.65 bc | 33.81cd |
| G1KL_5_ | 44.24 de | 15.77 d | 7.71 | 10.27 cd | 36.65 c | 36.18 bc | 30.67 bc | 34.50bc |
| G1KL_6_ | 51.27 b | 16.13 cd | 7.98 | 11.37 b | 37.00 bc | 36.55 b | 30.90 bc | 34.82b |
| G1KL_7_ | 51.87 b | 17.00 c | 8.14 | 11.43 b | 37.43 b | 36.80 b | 31.32 b | 35.18b |
| G2 KL_1_ | 52.53 b | 18.77 b | 6.89 | 10.60 c | 35.67 d | 34.95 cde | 30.47 bcd | 33.70cd |
| G2 KL_2_ | 33.73 h | 8.80 k | 4.50 | 5.37 k | 28.22 i | 27.33 j | 26.15 h | 27.23i |
| G2 KL_3_ | 33.93 h | 9.70 j | 5.14 | 6.37 j | 30.02 h | 29.05 i | 28.57 g | 29.21h |
| G2 KL_4_ | 41.53 fg | 12.20 h | 5.44 | 6.73 ij | 32.03 g | 31.73 h | 28.62 g | 30.79g |
| G2 KL_5_ | 42.60 efg | 13.67 g | 5.57 | 7.20 i | 32.30 g | 32.30 gh | 29.18 fg | 31.26fg |
| G2 KL_6_ | 46.37 cd | 14.77 ef | 5.87 | 8.23 gh | 33.60 f | 33.40 fg | 29.27 efg | 32.09ef |
| G2 KL_7_ | 48.27 c | 15.27 de | 6.02 | 9.62 de | 33.67 f | 33.58 efg | 29.50d-g | 32.25e |
| CV (%) | 4.26 | 5.18 | 9.28 | 6.67 | 1.85 | 3.51 | 2.99 | 2.41 |
| LSD (0.05) | 2.24 | 0.87 | 0.73 | 0.70 | 0.74 | 1.38 | 1.04 | 0.92 |
| LS | *** | * | ns | * | * | * | * | * |

. KL_1_ = WW + RKF (18 kg K ha^-1^); KL_2_ = WS + RKF; KL_3_ = WS + RKF + 25% additional K; KL_4_ = WS + RKF + 50% additional K; KL_5_ = WS + RKF + 75% additional K; KL_6_ = WS + RKF + 100% additional K; KL_7_ = WS + RKF + 125% additional K; G1= BMX-08010-2; G2= BARI Mung-1; LS = level of significance; * = significant at p-0.05; *** = significant at p-0.001

**Supplementary Table S5. Combined effects of additional K fertilization and mungbean genotypes on the Seed yield (average of 2018 and 2019).**

| Interactions | Seed yield (kg ha^-1^) | | | |
| --- | --- | --- | --- | --- |
|  | 1^st^ picking | 2^nd^ picking | 3^rd^ picking | Average |
| G1KL_1_ | 314.68 f | 571.48 a | 625.54 a | 1511.70a |
| G1KL_2_ | 234.46 h | 347.70 e | 378.84 e | 961.01ghi |
| G1KL_3_ | 250.85 gh | 371.48 cde | 372.85 e | 995.17fgh |
| G1KL_4_ | 262.73 g | 355.60 de | 426.50 d | 1044.83ef |
| G1KL_5_ | 270.57 g | 376.33 cd | 405.58 d | 1052.48e |
| G1KL_6_ | 253.67 gh | 382.43 c | 489.03 b | 1125.14d |
| G1KL_7_ | 264.68 g | 412.04 b | 508.20 b | 1184.93c |
| G2 KL_1_ | 602.76 a | 249.39 f | 456.89 c | 1309.04b |
| G2 KL_2_ | 400.31 e | 134.12 h | 154.91 h | 689.33k |
| G2 KL_3_ | 511.21 cd | 187.44 g | 196.65 g | 895.30j |
| G2 KL_4_ | 509.24 cd | 174.25 g | 248.15 f | 931.65ij |
| G2 KL_5_ | 500.15 d | 194.01 g | 253.53 f | 947.69hij |
| G2 KL_6_ | 527.20 c | 191.24 g | 271.15 f | 989.60gh |
| G2 KL_7_ | 555.13 b | 189.76 g | 257.17 f | 1002.06efg |
| CV (%) | 5.66 | 7.28 | 5.81 | 4.45 |
| LSD (0.05) | 25.71 | 25.07 | 24.41 | 54.16 |
| LS | *** | *** | *** | *** |

KL_1_ = WW+ RKF (18 kg K ha^-1^); KL_2_ = WS + RKF; KL_3_ = WS + RKF + 25% additional K; KL_4_ = WS + RKF + 50% additional K; KL_5_ = WS + RKF + 75% additional K; KL_6_ = WS + RKF + 100% additional K; KL_7_ = WS + RKF + 125% additional K; G1= BMX-08010-2; G2= BARI Mung-1; LS = level of significance; *** = significant at p-0.001

**Supplementary Table S6. Combined effects of additional K fertilization and mungbean genotypes on the stover and seed nutrient content (average of 2018 and 2019).**

| Interactions | Stover nutrient content | | | | Seed nutrient content | | | |
| --- | --- | --- | --- | --- | --- | --- | --- | --- |
|  | N (%) | P (%) | K (%) | S (%) | N (%) | P (%) | K (%) | S (%) |
| G1KL_1_ | 0.96 | 0.18 | 1.63 | 0.21 | 9.11 | 0.24 | 2.15 | 0.56 |
| G1KL_2_ | 0.71 | 0.17 | 1.05 | 0.14 | 6.63 | 0.20 | 1.60 | 0.42 |
| G1KL_3_ | 0.72 | 0.170 | 1.08 | 0.15 | 6.95 | 0.21 | 1.62 | 0.44 |
| G1KL_4_ | 0.77 | 0.17 | 1.16 | 0.16 | 7.45 | 0.22 | 1.69 | 0.50 |
| G1KL_5_ | 0.81 | 0.16 | 1.19 | 0.17 | 7.68 | 0.22 | 1.73 | 0.52 |
| G1KL_6_ | 0.88 | 0.16 | 1.31 | 0.17 | 8.51 | 0.24 | 1.85 | 0.56 |
| G1KL_7_ | 0.91 | 0.16 | 1.42 | 0.20 | 8.65 | 0.25 | 1.98 | 0.58 |
| G2 KL_1_ | 0.86 | 0.16 | 1.57 | 0.21 | 8.30 | 0.22 | 1.88 | 0.54 |
| G2 KL_2_ | 0.60 | 0.15 | 0.82 | 0.12 | 5.74 | 0.18 | 1.36 | 0.38 |
| G2 KL_3_ | 0.66 | 0.15 | 0.84 | 0.12 | 6.42 | 0.19 | 1.39 | 0.42 |
| G2 KL_4_ | 0.68 | 0.15 | 1.03 | 0.14 | 6.54 | 0.20 | 1.58 | 0.45 |
| G2 KL_5_ | 0.69 | 0.15 | 1.07 | 0.17 | 6.67 | 0.20 | 1.62 | 0.51 |
| G2 KL_6_ | 0.70 | 0.15 | 1.22 | 0.18 | 6.80 | 0.22 | 1.74 | 0.52 |
| G2 KL_7_ | 0.76 | 0.15 | 1.35 | 0.18 | 7.55 | 0.23 | 1.89 | 0.54 |
| CV (%) | 7.41 | 10.64 | 11.03 | 14.84 | 8.96 | 16.87 | 11.94 | 11.14 |
| LSD (0.05) | 0.07 | 0.02 | 0.15 | 0.03 | 0.77 | 0.04 | 0.24 | 0.06 |
| LS | ns | ns | ns | ns | ns | ns | ns | ns |

KL_1_ = WW + RKF (18 kg K ha^-1^); KL_2_ = WS + RKF; KL_3_ = WS + RKF + 25% additional K; KL_4_ = WS + RKF + 50% additional K; KL_5_ = WS + RKF + 75% additional K; KL_6_ = WS + RKF + 100% additional K; KL_7_ = WS + RKF + 125% additional K; G1= BMX-08010-2; G2= BARI Mung-1; LS = level of significance; ns = nonsignificant
